# MR molecular imaging of tumors based on an optimal hTERT promoter tyrosinase expression system

**DOI:** 10.18632/oncotarget.9888

**Published:** 2016-06-07

**Authors:** Chuan Li, Chang-Jiang Hu, Bo Tang, Xin Yong, Gang Luo, Yu-Yun Wu, Su-Min Wang, Song-Tao Yu, Shi-Ming Yang

**Affiliations:** ^1^ Department of Radiology, Southwest Hospital, Third Military Medical University, Chongqing 400038, P.R. China; ^2^ Department of Gastroenterology, Xinqiao Hospital, Third Military Medical University, Chongqing 400037, P.R. China; ^3^ Department of Oncology, Southwest Hospital, Third Military Medical University, Chongqing 400038, P.R. China

**Keywords:** MR molecular imaging, hTERT promoter, tyrosinase, melanin

## Abstract

The early diagnosis and treatment of tumors is of vital significance to increase patient survival. Therefore, we constructed a lentiviral vector expressing tyrosinase (TYR) driven by an optimized human telomerase reverse transcriptase (hTERT) promoter or a cytomegalovirus(CMV) promoter in the hopes of performing noninvasive and real-time tumor-specific imaging. First, hTERT-TYR and CMV-TYR were constructed to infect cancer cell lines (telomerase-negative cell line: U2OS; telomerase-positive cell lines: SGC-7901, SW480 and HepG2). Subsequently, stable tyrosinase-expressing cell lines were sorted by flow cytometry out of these infected cancer cell lines. Then, the mRNA and protein levels of tyrosinase were analyzed. Thetyrosinase activity, melanin production and ferric ion adsorption were measured followed by an MR scan. Consequently the results showed that tyrosinase was only expressed in telomerase-positive tumor cells infected by hTERT-TYR, whereas tyrosinase was expressed in both telomerase-negative and telomerase-positive tumor cells infected by CMV-TYR. Finally, we performed in vivo tumor MR using a clinical 3T MR scanner and found increased signals at T1W1 from telomerase-positive cells infected by hTERT-TYR, which revealed that MR scanning could distinguish cells with hTERT -positive cells from hTERT-negative cells infected with the optimized lentivirus. The mechanism underlying this effect is that tyrosinase promotes melanin production and ferric ion adsorption only in hTERT-expressing cells. Taken together, these data show that this optimized hTERT promoter-driving tyrosinase expression system might be a useful diagnostic tool for the detection of tumors using MR imaging.

## INTRODUCTION

With new developments in molecular biology and applications of molecular probes, MR molecular imaging has provided new ideas for the diagnosis of tumors [1]. Many researchers have discussed the concept of using tyrosinase (TYR) as an MR reporter gene [2, 3, 4, 5]. TYR is introduced into cells through gene transfection. The expressed tyrosinase then catalyzes the synthesis of melanin from tyrosine precursors, which can induce the absorption of extracellular and intracellular metal cations, especially Fe^3+^ [6]. As a paramagnetic material, the concentration of Fe^3+^ closely correlates with changes in the MR TIWI signal. The targeted tissue can be specifically displayed with high intensity on T1WI [3]. Therefore, if the TYR gene can be driven by specific promoters that are active in tumor cells but not in normal tissues or cells, it could be used as an MR readout for tumors. Human telomerase reverse transcriptase (hTERT), as the rate-limiting enzyme of telomerase, plays an important role in the regulation of its activity [7]. It has been reported that hTERT could be used as both a unique tumor marker and an effective anticancer target [8, 9, 10]. Similarly, the hTERT promoter targets tumors especially and can therefore be considered a tumor-specific promoter and used for the targeted diagnosis and treatment of tumors [11]. It has been reported that adding three Myc-binding E-box (CACGTG) motifs to the hTERT promoter improved its tumor targeting effect [12, 13]. In our previous studies, we successfully designed and constructed an optimized hTERT promoter, which contained the core region of the hTERT promoter and three Myc-binding motifs [14]. The cytosine deaminase (CD) gene driven by the optimized hTERT promoter significantly improved the suicidal effect of the hTERT-positive tumor cells [15]. In this study, we first constructed a recombinant tyrosinase expression plasmid driven by the optimized hTERT promoter to test the signal of xenograft tumors in vivo using MR scanning.

## RESULTS

### hTERT-driven expression of tyrosinase was observed only in hTERT-positive cells

**Figure 1:**
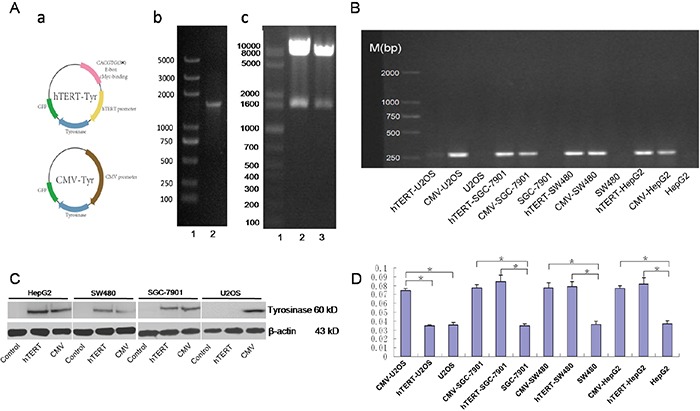
hTERT-driven expression of the tyrosinase system is functional in hTERT-positive cells **A.** Construction of a reconstructed tyrosinase expression plasmid driven by the hTERT or CMV promoter. a. Schematic diagram of the constructed plasmid. b. TYR DNA was amplified by PCR with primers ranking the TYR open reading frame. c. phTERT/TYR-IRES2-EGFP (lane 2) and pCMV/TYR-IRES2-EGFP plasmids (lane 3) were digested with both EcoR I and Sal I. **B** and **C.** PCR and Western blot were used to detect expression of tyrosinase in various cell lines. Three hTERT-positive cell lines (HepG2, SGC-7901 and SW480) and one hTERT-negative cell line (U2OS) were infected with hTERT-TYR and CMV-TYR, respectively. Then, PCR and Western blot were used to assay the expression of tyrosinase mRNA (B) and protein (C). **D.** Tyrosinase activity in the four cell lines described above was assessed using the method described in Korner et al (30) and read out as the absorbance at 490 nm.

TYR DNA was amplified by PCR and then cloned into an hTERT-pIRES2-EGFP plasmid (Figure 1A a-b). A CMV-pIRES2-EGFP plasmid was used as a positive control. The recombinant plasmids were successfully digested with EcoR I and Sal I (Figure 1 Ac). They were then packaged into the lentivirus. To test the selective expression of hTERT-TYR, three hTERT-positive cell lines (SGC-7901, SW480 and HepG2) were infected with the lentiviral constructs, as was an hTERT negative cell line (U2OS) that served as a negative control. Compared with the corresponding control (uninfected cells), the CMV promoter drove tyrosinase gene expression in both hTERT-positive and negative cell lines, as shown by an analysis of the mRNA (Figure 1B) and protein (Figure 1C) levels. The tyrosinase activity in these cells mimicked the mRNA and protein levels (Figure 1D). These results demonstrate that hTERT-TYR selectively drives tyrosinase expression and activity in hTERT-positive tumor cells.

### hTERT-TYR selectively promotes melanin production in hTERT-positive tumor cells

**Figure 2:**
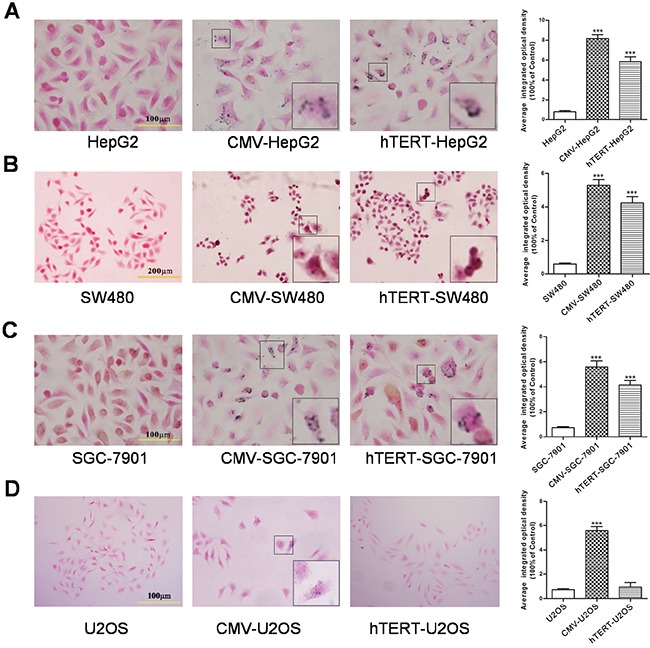
hTERT-TYR selectively promotes melanin production in hTERT-positive tumor cells U2OS, HepG2, SGC7901 and SW480 cells infected with hTERT-TYR or CMV-TYR were seeded into six-well plates at a density of 1×10^5^ per well. Seventy-two hours later, cells were harvested for Fontana staining to analyze the production of melanin. Uninfected cells were used as a control. Fontana staining of **A.** HepG2 cells, **B.** SW480 cells, **C.** SGC-7901 cells and D. U2OS cells.

Because tyrosinase is the key enzyme for the production of melanin, the amount of melanin in cells could serve as a readout for tyrosinase activity. Therefore, we performed Fontana staining to examine melanin levels. As shown in Figure 2, compared with the corresponding control group, hTERT-TYR enhanced melanin production in hTERT-positive cell lines (SGC-7901, SW480 and HepG2) but not in hTERT-negative lines (U2OS), whereas CMV-TYR increased the amount of melanin in all cell lines. These results clearly demonstrate that the hTERT-TYR system has high activity in cells with high expression levels of hTERT.

### hTERT-TYR selectively promotes adsorption of ferric ions in hTERT-positive cells

**Figure 3:**
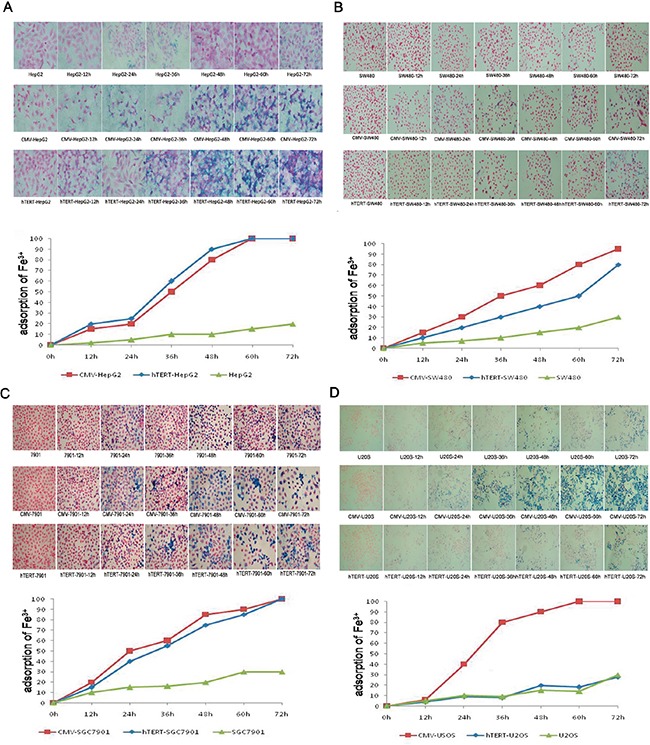
hTERT-TYR selectively promotes adsorption of ferric ions in hTERT-positive cells U2OS, HepG2, SGC7901 and SW480 cells infected with hTERT-TYR or CMV-TYR were seeded into 24-well plates at a density of 1×10^4^ per well. Subsequently, the cells were incubated with 5 mg/μl iron ions for various lengths of time and then subjected to Prussian blue staining to assay the adsorption of the ferric ions in different cells. Uninfected cells were used as a control. **A-D.** Prussian blue staining and corresponding quantitation results of the four cell lines.

As shown in Figure 3A-3D, compared with the corresponding control group, hTERT-TYR enhanced the adsorption of ferric ions in hTERT-positive cell lines (SGC-7901, SW480 and HepG2) but not in the hTERT-negative cell line (U2OS), whereas CMV-TYR increased the adsorption of ferric ions in all of the cell lines. These results further demonstrate that the adsorption of ferric ions induced by hTERT-TYR was increased in hTERT-expressing cells.

### MR scanning reflects the selective activity of hTERT-TYR in cells with high hTERT expression in vitro

**Figure 4:**
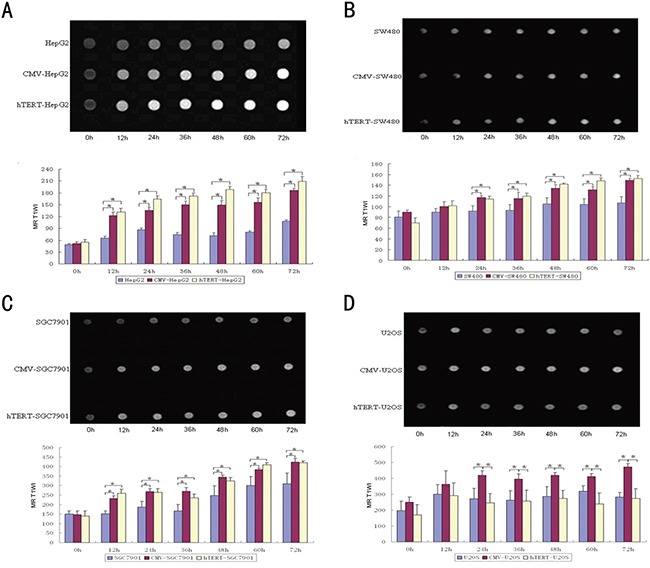
MR scanning reflects the selective expression activity of hTERT-TYR in hTERT-expressing cells After incubation of 7×10^7^ of each of the cells mentioned above with 5 mg/μl ferric ions for various amounts of time (from 12 to 72 h), cells were harvested and centrifuged for MR scanning at T1WI. **A-D.** The MR T1WI signal and corresponding quantitative signal results of the cells at various time points.

Ferric ions show a specific, high signal at MR T1WI that is positively associated with the level of ferric ions. As shown in Figure 4A-4D, in the hTERT-TYR group, the signal at MR T1WI increased in a time-dependent manner in hTERT-positive cells (especially HepG2 cells and SGC-7901) but not U2OS cells. However, the MR T1WI signal of all the CMV-TYR cells increased. These results suggest that MR scanning based on the hTERT-driven tyrosinase system can specifically identify cells with high hTERT expression.

### MR scanning reflects the selective activity of hTERT-TYR in hTERT-expressing cells in vivo

**Figure 5:**
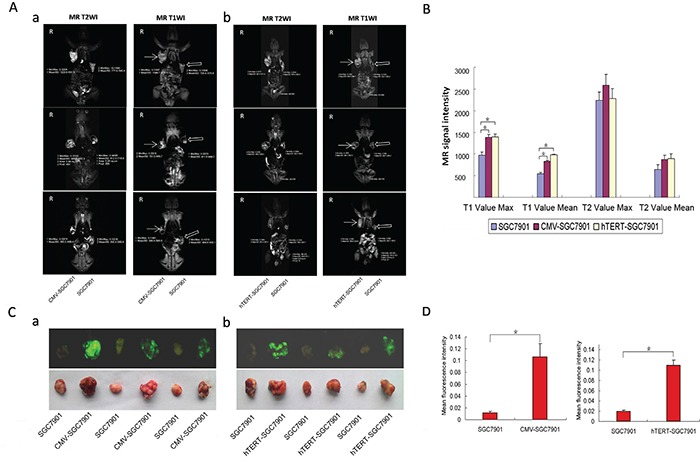
MR scan of xenografted tumors derived from SGC-7901 cells infected with various lentiviral constructs **A.** MR scans of various xenograft tumors in vivo. Severe combined immune deficiency (SCID) mice (4-5 weeks old) were s.c. injected with 1×10^6^ cells (control group) on the left side and pCMV/TYR-IRES2-EGFP or phTERT/TYR-IRES2-EGFP cells (SGC-7901 cells) on the right side. All mice were supplied with 130 μl 1 mg/ml ferric ions by intraperitoneal injection (once every three days for nine days). Four weeks after the last injection, mice were anesthetized and scanned by MR TIWI and T2WI. **B.** The maximums and averages of the signals for MR T1WI and MR T2WI. **C.** Harvested tumors were also imaged under white light using an invivo Multispectral Imaging System. **D.** The quantified results of tumor fluorescence imaging.

SCID mice injected with various types of cells were subjected to MR scanning. As shown in Figure 5A, the CMV-SGC-7901 group (Figure 5Aa) showed a higher signal at MR T1WI compared to the control, and similar results were obtained in the hTERT-SGC-7901 group (Figure 5Ab). However, MR T2WI scanning did not show differences in the signal levels between the groups (Figure 5A). An in vivo Multispectral Imaging System was used to image GFP expression in the mice, and the tumors were also harvested and imaged separately. The maximums and averages of the signal strengths at MR T1WI and MR T2WI were calculated and are shown in Figure 5B. This result further demonstrates that MR T1WI but not MR T2WI reflects the signal change caused by the reporter lentivirus in cells with varying levels of hTERT expression. As shown in Figure 5C, both the CMV-SGC-7901 group and the hTERT-SGC-7901 group had higher green fluorescence signals than the control group, which was consistent with the results in Figure 5A. The results shown in Figure 5C are quantified in Figure 5D. Quite consistent results were observed in xenograft tumors derived from HepG2 cells (Supplementary Figure 1). On the contrary, the MR T1WI signal was unchanged in the hTERT-U2OS group compared with the corresponding control group (U2OS; Supplementary Figure 2). These results imply that MR T1WI can be used to distinguish hTERT-positive tumors from the hTERT-negative tumors using the hTERT-TYR system.

### hTERT-TYR selectively promotes melanin production and adsorption of ferric ions in xenografted tumor

**Figure 6:**
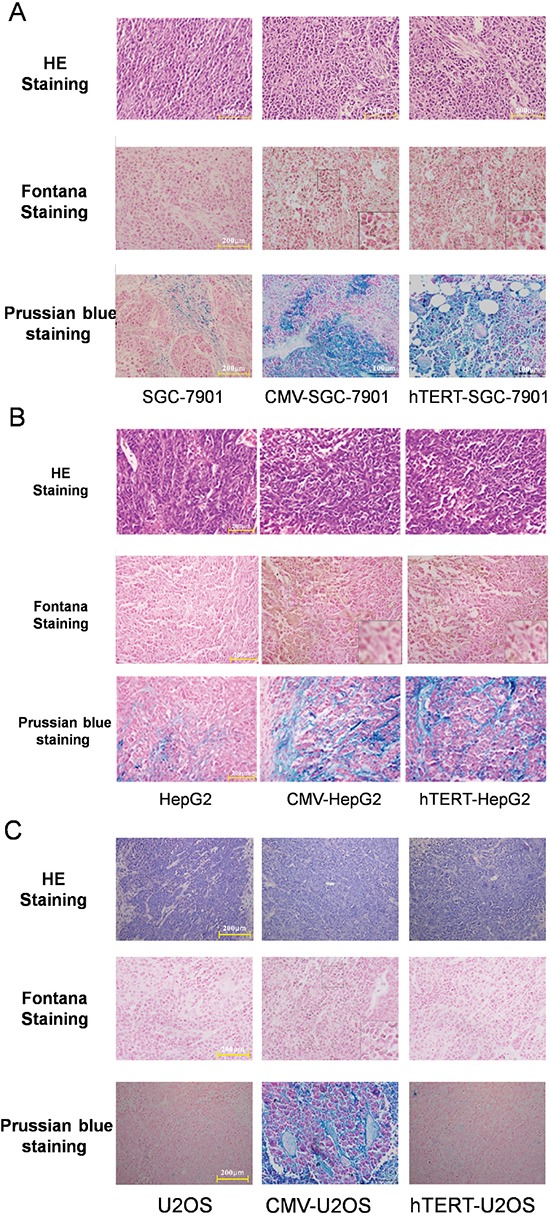
Melanin production and adsorption of ferric ions in xenograft tumors derived from various types of cells **A.** SGC-7901 cells. **B.** HepG2 cells. **C.** U2OS cells.

Above, we showed that MR T1WI can reflect the expression of the reporter lentivirus in cells with varying hTERT expression (Figures 5, Supplementary 1 and 2) in vivo. Fontana staining and Prussian blue staining were further employed to test melanin production and adsorption of ferric ions in xenografted tumors (Figure 6). The H&E staining revealed the tumor organizational structure. Fontana staining showed that hTERT-TYR and CMV-TYR increased melanin production in SGC7901 and HepG2 cells, whereas both of them induced chelation of ferric ions (Figure 6A, 6B). Interestingly, CMV-TYR but not hTERT-TYR promoted melanin production and adsorption of ferric ions in U2OS cells (Figure 6C).

## DISCUSSION

In this study, we successfully demonstrated that a TYR gene for MR imaging combined with the tumor target of an hTERT promoter can be used to diagnose tumors in vitro and in vivo. Supplementary Figure 3 schematically illustrates the principle of targeted cancer MR molecular imaging. The reasons are described below.

First of all, TYR, as an endogenous gene, is the modulator of the synthesis of melanin, which is present in many human pigmented cells and tissues. It catalyzes the hydroxylation of tyrosine-yielding dioxyphenylalanine (DOPA) and its subsequent oxidation to DOPA quinone, which can be converted into melanin [16]. Melanin has a high affinity for metal ions, including Fe^3+^, Mg^2+^ and Ca^2+^, and thus can serve as a transporter of metal ions due to the many channels within melanin granules. There are approximately 10^20^ ion adsorption sites for every gram of melanin [17, 18]. It can absorb up to 35% of its own weight in iron particles [19]. Due to the paramagnetic effects of iron, its accumulation substantially shortens the T1 relaxation time around the tissue of the melanin, which will give rise to a high signal level on MR T1WI. In fact, clinical observations have confirmed that melanotic melanomas are associated with hyperintensity on T1-weighted MR images [20, 21]. Thus, melanin caused by the TYR gene can be exploited as a contrast mechanism in MR molecular images [22].

However, prior studies using melanin as an MR contrast agent were mostly carried out in cells. Therefore, an in vivo proof-of-concept study is important [3, 23]. In our work, we have successfully demonstrated that hTERT-TYR can be applied as an MR reporter gene both in vitro and in vivo, and excellent tumor target imaging has been achieved.

It is of great importance to guarantee that reporter genes are only expressed in most tumor cells instead of normal cells. It is widely known that many commonly used promoters can regulate the expression of reporter genes. However, some promoters, such as cytomegalovirus and Simian virus 40, do not have tumor specificity, whereas others, such as alpha-fetoprotein, carcinoembryonic antigen and prostate-specific antigen promoters, show strong specificity only in certain tumors.

The human telomerase complex, known as the human telomerase catalytic subunit (hTERT), has been found to be expressed in the majority of tumors of all cancer types [24, 25], including cancer stem cells [26]. Also, the abnormally high expression of hTERT leads to carcinogenesis [27]. In our previous study, we designed an optimized hTERT promoter by insertion of several copies of a c-myc binding sequence, and the results suggested that the optimized promoter showed strict telomerase specificity [14, 15]. In this study, we still used the same optimized telomere as the specific hTERT promoter to drive TYR gene expression to diagnose tumors.

According to Massoud [28] et al, the sensitivity of MR probe detection is 10^6^-10^9^ times lower than PET and 10^10^-10^14^ times lower than bioluminescence. Therefore, EGFP was used as a positive control to evaluate the feasibility and sensibility of TYR as the MR reporter gene in our study. We constructed a dual reporter gene system in which EGFP and TYR were cloned downstream of the promoter by inserting an internal ribosomal entry site sequence between the two genes, and they were transcribed into a single messenger RNA and later translated into two different proteins. A strong promoter, CMV, was used as the positive control to verify the detection of hTERT promoter activity. Besides, gene transfer systems are a critical factor in guaranteeing the successful tumor specificity visualization. In our study, a lentivirus-mediated transgene expression system was applied to improve gene transfection efficiency because lentiviral vectors can integrate target genes into the host genome to achieve long-term stable expression.

In summary, we constructed a lentivirus co-expressing EGFP and TYR genes driven by an optimized hTERT promoter and CMV promoter, respectively. Fontana staining showed that hTERT-TYR selectively promoted melanin production in hTERT-positive tumor cells but not in hTERT-negative tumor cells. Prussian blue staining revealed that the optimized hTERT-TYR dramatically promoted the adsorption of ferric ions in hTERT-positive tumor cells but not in hTERT-negative tumor cells. Moreover, in vitro and in vivo experiments showed that hTERT-positive tumor cells infected with hTERT-TYR were detectable with a specific, high signal at TIWI in an MR scan. All the results demonstrated that hTERT-TYR can be specifically expressed in telomerase-positive tumor cells, and the activity of this optimized hTERT promoter was found to be equal to the activity of CMV promoters in vitro and in vivo.

Comparing hTERT-positive tumor cells infected by hTERT-TYR with ones free from infection, the results showed that the signal of T1WI MR images slightly increased when the fluorescence intensity was obviously improved by the CCD imaging system. The hTERT-TYR gene as MR reporter has comparatively lower sensitivity than the EGFP gene in CCD imaging, but hTERT is commonly recognized as an effective anticancer target and hTERT-TYR MR imaging has the advantage that the specific signal can be co-registered with soft-tissue anatomy and functional tissue information to clearly define and demonstrate the size, the range and the internal structures of the tumors. Collectively, this technology provides a valuable and noninvasive method for tumor diagnosis. We expect this work will stimulate further studies on hTERT-TYR to diagnose tumors.

## MATERIALS AND METHODS

### Construction of lentiviral vectors for qualitative tumor diagnosis

First, TYR DNA was amplified by PCR with primers ranking the TYR open reading frame with EcoRI and Sal I restriction enzyme sequences within the 5′and 3′primers, respectively. The purified TYR DNA and the hTERTp/CD-IRES2-EGFP and CMVp/CD-IRES2-EGFP (constructed in our previous study [15]) plasmids were digested with EcoRI and Sal I restriction enzymes (New England Biolabs, Inc., Ipswich MA, USA) and ligated together with DNA ligase (New England Biolabs). The ligation mixture was used to transform E.coli DH5a competent cells, and a large amount of the recombinant plasmids were acquired. Subsequently, they were packaged into lentivirus and referred to as hTERT-TYR and CMV-TYR as described previously [14].

### Infection and sorting of stable tyrosinase-expressing cell lines

Four human malignant tumor cell lines were used in this study. Telomerase-positive cell lines HepG2, SGC-7901, SW480(purchased from the Institute of Biochemistry and Cell Biology) and telomerase-negative cell line U2OS(from the American Type Culture Collection) were seeded into six-well plates at a density of 1×10^5^ per well in duplicate. Twenty-four hours later, CMV-TYR and hTERT-TYR were added into each pair of wells at an optimal MOI for each lentivirus. Seventy-two hours after infection, GFP-positive cells were sorted by flow cytometry (BD Biosciences, San Jose, CA, USA). The infected cells were named CMV-HepG2, hTERT-HepG2, and so on.

### Reverse-transcription PCR and western blot for tyrosinase expression

TYR expression was further confirmed by reverse-transcription PCR (RT-PCR) for TYR messenger RNA (mRNA) and Western blotting for TYR protein. The primers for TYR were 5′-GAATTC ATGCTCCTGGCTGTTTTGTACTG-3′ (forward primer) and 5′-GTCGACTAAATGGCTCTGATACAAGCTGTGG-3′ (reverse primer); β-actin was amplified using the forward primer 5′-CTTCTACAATGAGCTGCGTG-3′ and the reverse primer 5′-TCATGAGGTAGTCAGTCAGG-3′. Western blot analysis was performed as described previously [29]. The primary antibody to anti-tyrosinase (sc-73243) was from Bioworld Technology (Minneapolis, MN, USA), and the anti-β-Actin antibody (sc-47778) was from Santa Cruz Biotechnology (Santa Cruz, CA).

### Measurement of tyrosinase activity in tumor cell lines

Tyrosinase activity was assessed using the method described by Korner et al [30]. Briefly, the 12 cell lines mentioned above were seeded into 96-well plates at a density of 1×10^4^ per well. Twenty-four hours later, the cells were washed, lysed with 1% Triton-X-100 (50 μl/well) and frozen at −80°C for 30 min. The cells were allowed to rupture completely at room temperature. Following the addition of 10 g/L-DOPA into the wells at 37°C for 2 hours, the absorbance value was measured at 490 nm to detect tyrosinase activity.

### Fontana staining for melanin production

Uninfected U2OS, HepG2, SGC7901 and SW480 cells, as well as those infected with CMV-TYR or hTERT-TYR, were seeded into six-well plates at a density of 1×10^5^ per well. Seventy-two hours later, the culture medium was removed, the cells were washed with PBS, and each slide was fixed in paraformaldehyde for 20 minutes followed by Fontana staining. Briefly, the slides were soaked in gold chloride, placed in Hypo and in nuclear fast red and dehydrated.

### Prussian blue staining for ferric ion adsorption

The cells described above were seeded into 24-well plates at a density of 1×10^4^ per well. Subsequently, the cells were incubated with 5 mg/μl iron ions for various lengths of time. Then, the slides were fixed in 4% paraformaldehyde, washed, incubated for 30 min with 2% potassium ferrocyanide (Perls' reagent) in 6% HCl, washed, and counterstained with nuclear fast red.

### MR scan for signal changes of the tumor cell lines in vitro

After the 7×10^7^ cells described above were incubated with 5 mg/μl ferric ions for various amounts of time (from 12 h to 72 h), they were harvested and centrifuged before MR scanning. MR images of the cell samples were obtained in a clinical 3T MR scanner (Siemens, Germany).A*T*1-weighted, 2D Turbo spin echo (TSE) acquisition sequence and a 16-channel head phased-array coil were used for detection during high-resolution imaging. The imaging parameters were a TR of 46 ms, a TE of 6.5 ms, a flip angle of 65°, a matrix of 256×256, a field of view (FOV) of 55 mm, a slice thickness of 2 mm, and an average of 5.

### In vivo tumor MR imaging and fluorescence imaging

Severe combined immune deficiency (SCID) mice (4-5 weeks old) were purchased from Third Military Medical University (Chongqing, China). For injection of each cell line (HepG2, U2OS and SGC-7901), the mice were divided into two groups (3 mice per group). In one group, mice were injected s.c. with 1×10^6^ cells (control group) on the left side and CMV-TYR cells on the right side. In another group, mice were injected with 1×10^6^ cells s.c. (control group) on the left side and hTERT-TYR cells on the right side. One week later, all mice were given 130 μL 1 mg/ml ferric ions by intraperitoneal injection (once every three days for nine days). Four weeks after the injection, the mice were anesthetized and scanned by MR. They were also imaged using a KODAK In-Vivo Multispectral Imaging System FX (Carestream Health) with an exposure time of 10 s and a standard excitation spectrum of 470 nm, which permits an emission spectrum of 535 nm and a field of view of 150 mm. Finally, the mice were sacrificed, and the tumors were dissected for fluorescence imaging to confirm the transgene expression in tumor tissue. Moreover, histology experiments using hematoxylin-eosin (H&E), Fontana and Prussian blue staining were performed.

### Statistical analysis

All data are expressed as the means ± standard deviations (SD). Comparisons between three or more groups were made using an ANOVA followed by Tukey–Kramer post hoc analysis. In all cases, a difference was considered statistically significant if the *p* value was <0.05 (“*” represents *p*<0.05).

## SUPPLEMENTARY FIGURES



## Abbreviations

hTERT: human telomerase reverse transcriptase
CT: computed tomography
MRI: magnetic resonance imaging
TYR: tyrosinase

## References

[1] McCann TE, Kosaka N, Turkbey B, Mitsunaga M, Choyke PL, Kobayashi H (2011). Molecular imaging of tumor invasion and metastases: the role of MRI. NMR Biomed.

[2] Weissleder R, Simonova M, Bogdanova A, Bredow S, Enochs WS, Bogdanov A (1997). MR imaging and scintigraphy of gene expression through melanin induction. Radiology.

[3] Enochs WS, Petherick P, Bogdanova A, Mohr U, Weissleder R (1997). Paramagnetic metal scavenging by melanin: MR imaging. Radiology.

[4] Isiklar I, Leeds NE, Fuller GN, Kumar AJ (1995). Intracranial metastatic melanoma: correlation between MR imaging characteristics and melanin content. AJR Am J Roentgenol.

[5] Vandsburger MH, Radoul M, Cohen B, Neeman M (2013). MRI reporter genes: applications for imaging of cell survival, proliferation, migration and differentiation. NMR Biomed.

[6] Samokhvalov A, Liu Y, Simon JD (2004). Characterization of the Fe(III)-binding site in Sepia eumelanin by resonance Raman confocal microspectroscopy. Photochem Photobiol.

[7] Poole JC, Andrews LG, Tollefsbol TO (2001). Activity, function, and gene regulation of the catalytic subunit of telomerase (hTERT). Gene.

[8] Eissenberg JC (2013). Telomeres, cancer & aging: live long & prosper?. Mo Med.

[9] Yang SM, Fang DC, Yang JL, Chen L, Luo YH, Liang GP (2008). Antisense human telomerase reverse transcriptase could partially reverse malignant phenotypes of gastric carcinoma cell line in vitro. Eur J Cancer Prev.

[10] Guo H, Hao J, Wu C, Shi Y, Zhao XY, Fang DC (2007). A novel peptide-nucleotide dual vaccine of human telomerase reverse transcriptase induces a potent cytotoxic T-cell response in vivo. Biochem Biophys Res Commun.

[11] Kim NW, Piatyszek MA, Prowse KR, Harley CB, West MD, Ho PL, Coviello GM, Wright WE, Weinrich SL, Shay JW (1994). Specific association of human telomerase activity with immortal cells and cancer. Science.

[12] Horikawa I, Cable PL, Mazur SJ, Appella E, Afshari CA, Barrett JC (2002). Downstream E-box-mediated regulation of the human telomerase reverse transcriptase (hTERT) gene transcription: evidence for an endogenous mechanism of transcriptional repression. Mol Biol Cell.

[13] Liu C, Fang XL, Ge Z, Jalink M, Kyo S, Björkholm M, Gruber A, Sjöberg J, Xu D (2007). The telomerase reverse transcriptase (hTERT) gene is a direct target of the histone methyltransferase SMYD3. Cancer Res.

[14] Yu ST, Yang YB, Liang GP, Li C, Chen L, Shi CM, Tang XD, Li CZ, Li L, Wang GZ, Wu YY, Yang SM, Fang DC (2010). An optimized telomerase-specific lentivirus for optical imaging of tumors. Cancer Res.

[15] Yu S T, Li C, Lü MH, Liang GP, Li N, Tang XD, Wu YY, Shi CM, Chen L, Li CZ, Cao YL, Fang DC, Yang SM (2012). Noninvasive and real-time monitoring of the therapeutic response of tumors in vivo with an optimized hTERT promoter. Cancer.

[16] Jawaid S, Khan TH, Osborn HM, Williams NA (2009). Tyrosinase Activated Melanoma Prodrugs. Anticancer Agents Med Chem.

[17] Liu Y, Hong L, Kempf VR, Wakamatsu K, Ito S, Simon JD (2004). Ion-exchange and adsorption of Fe(III) by Sepia melanin. Pigm Cell Res.

[18] Mishima Y (1994). Molecular and biological control of melanogenesis through tyrosinase genes and intrinsic and extrinsic regulatory factors. Pigment Cell Res.

[19] Sarna T, Froncisz W, Hyde JS (1980). Cu2+ probe of metal-ion binding sites in melanin using electron paramagentic resonance spectroscopy II. Natural melanin. Arch Biochem Biophys.

[20] Ginat DT, Meyers SP (2012). Intracranial lesions with high signal intensity on T1-weighted MR images: differential diagnosis. Radiographics.

[21] Woodruff WW, Djang WT, McLendon RE, Heinz ER, Voorhees DR (1987). Intracerebral malignant melanoma: high-field-strength MR imaging. Radiology.

[22] Paproski RJ, Forbrich AE, Wachowicz K, Hitt MM, Zemp RJ (2011). Tyrosinase as a dual reporter gene for both photoacoustic and magnetic resonance imaging. Biomed Opt Express.

[23] Alfke H, Stoppler H, Nocken F, Heverhagen JT, Kleb B, Czubayko F, Klose KJ (2003). In vitro MR imaging of regulated gene expression. Radiology.

[24] Hiyama E, Hiyama K (2003). Telomerase as tumor marker. Cancer Lett.

[25] Shay JW, Bacchetti S (1997). A survey of telomerase activity inhuman cancer. Eur J Cancer.

[26] Armanios M, Greider CW (2005). Telomerase and cancer stemcells. Cold Spring Harb Symp Quant Biol.

[27] Kelland L (2007). Targeting the limitless replicative potential of cancer: the telomerase/telomere pathway. Clin Cancer Res.

[28] Massoud T F, Gambhir S S (2003). Molecular imaging in living subjects: seeing fundamental biological processes in a new light. Genes & development.

[29] Xiong Z, Lü MH, Fan YH, Cao YL, Hu CJ, Wu YY, Wang SM, Luo G, Fang DC, Li C, Yang SM (2012). Downregulation of heparanase by RNA interference inhibits invasion and tumorigenesis of hepatocellular cancer cells in vitro and in vivo. Int J Oncol.

[30] Korner A, Pawelek J (1982). Mammalian tyrosinase catalyzes three reactions in the biosynthesis of melanin. Science.

