# Supplementary material for: MR molecular imaging of tumors based on an optimal hTERT promoter tyrosinase expression system
Source: Oncotarget. 2016 Jun 7;7(27):42474–84. doi: 10.18632/oncotarget.9888 (PMC5173149; doi:10.18632/oncotarget.9888)
Supplement: Supplementary file 1 [file oncotarget-07-42474-s001.pdf]

SUPPLEMENTARY FIGURES

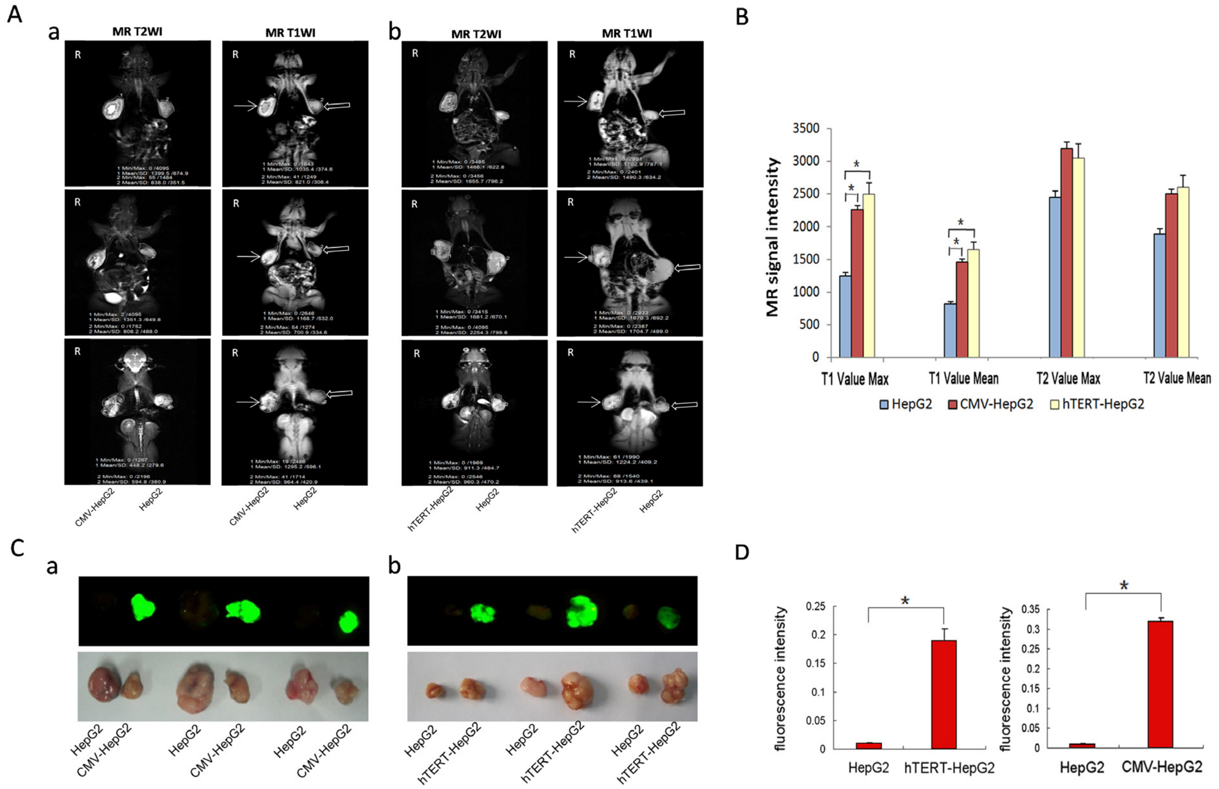

Supplementary Figure S1: MR scan of xenograft tumors derived from HepG2 cells infected with various lentiviral constructs. A. MR scan of the various xenograft rumors in vivo. Severe combined immune deficiency (SCID) mice (4-5 weeks old) were s.c. injected with 1×10^6 cells (control group) on the left side and pCMV/TYR-IRES2-EGFP or phTERT/TYR-IRES2-EGFP cells (HepG2 cells) on the right side. All mice were supplied with 130 µL 1 mg/ml ferric ions by intraperitoneal injection (once every three days for nine days). Four weeks after the last injection, mice were anesthetized and scanned by MR TIWI and T2WI. **B.** The maximums and averages of the signals for MR T1WI and MR T2WI. **C.** Harvested tumors were also imaged under white light using anin vivo Multispectral Imaging System. **D.** The quantified results of tumor fluorescence imaging.

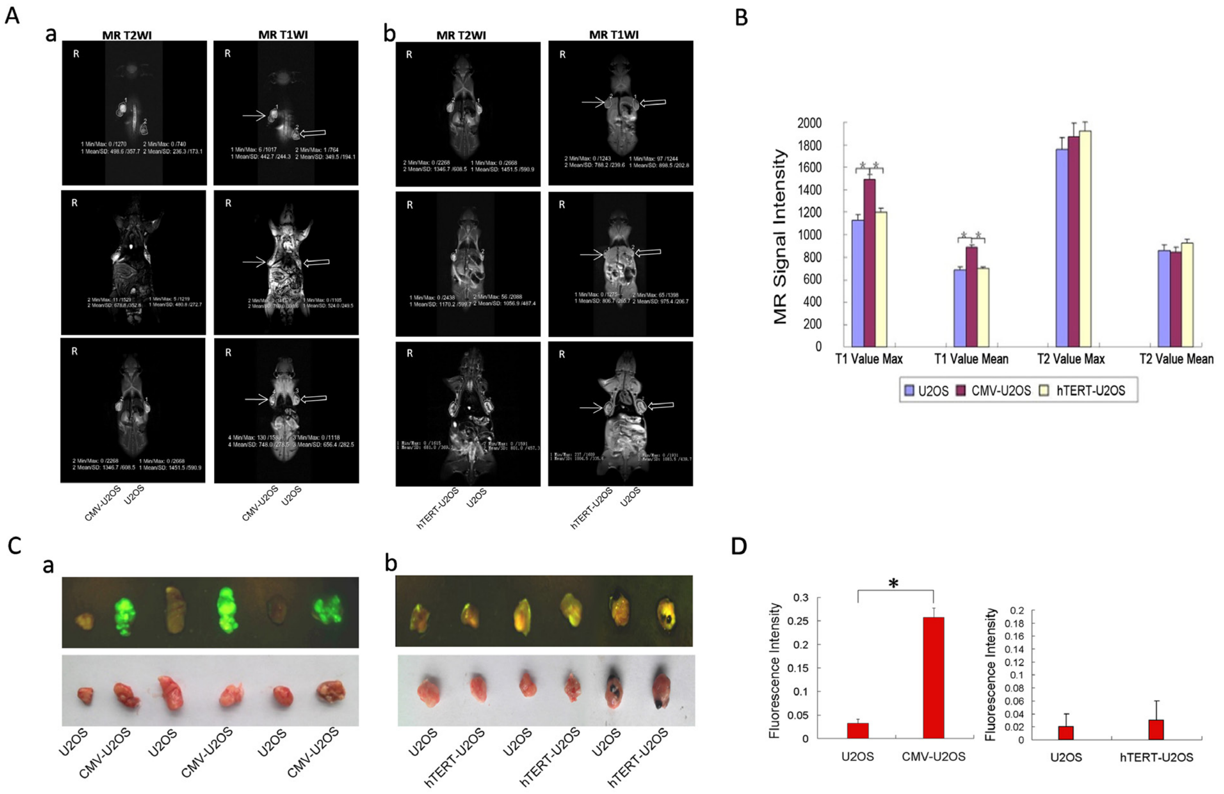

Supplementary Figure S2: MR scans of xenograft tumors derived from U2OS cells infected with various lentiviral constructs. A. MR scan of various xenograft tumors in vivo. Severe combined immune deficiency (SCID) mice (4-5 weeks old) were s.c. injected with 1×10^6 cells (control group) on the left side and pCMV/TYR-IRES2-EGFP or phTERT/TYR-IRES2-EGFP cells (U2OS cells) on the right side. All mice were supplied with 130 µL 1 mg/ml ferric ions by intraperitoneal injection (once every three days for nine days). Four weeks after the last injection, mice were anesthetized and scanned by MR TIWI and T2WI. **B.** The maximums and averages of the signals for MR T1WI and MR T2WI. **C.** Harvested tumors were also imaged under white light using aninvivo Multispectral Imaging System. **D.** The quantified results of tumor fluorescence imaging.

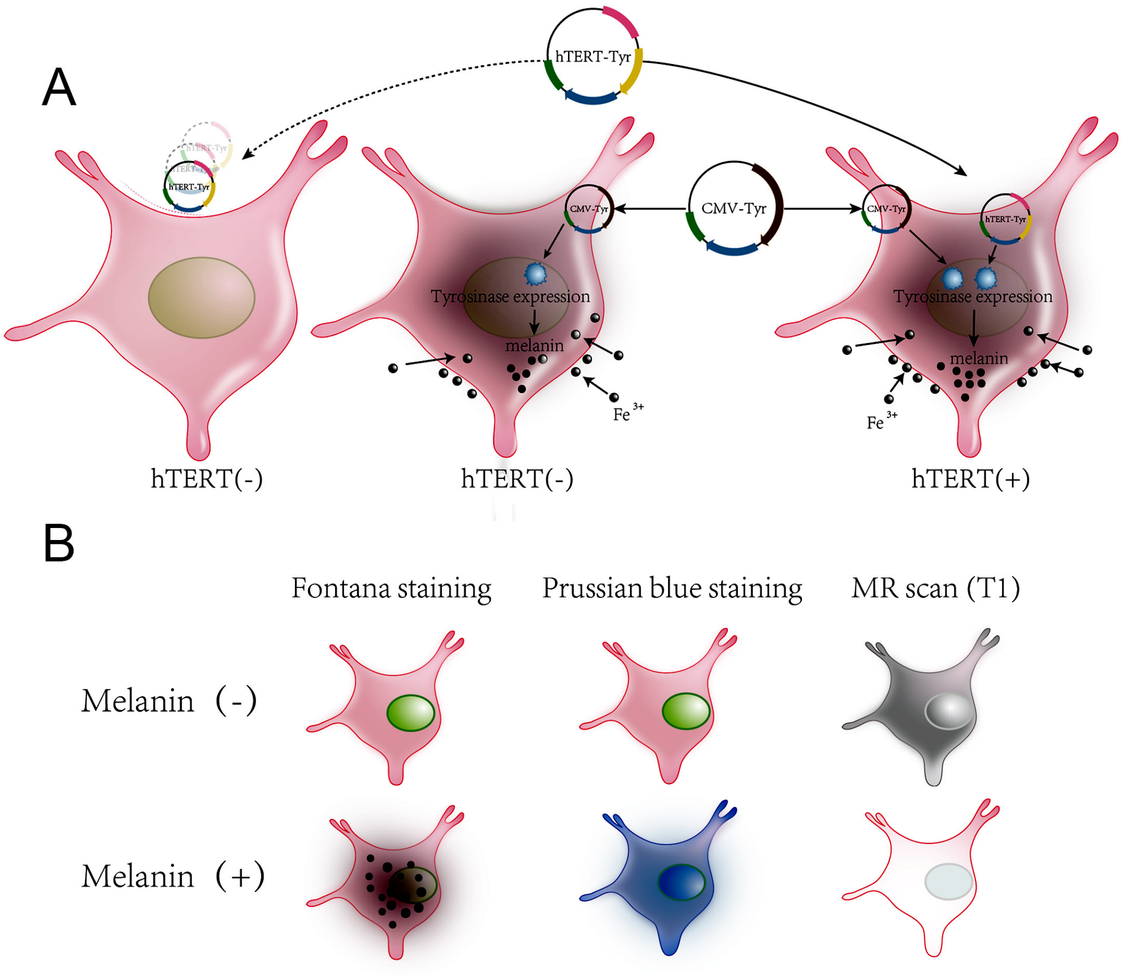

Supplementary Figure S3: Schematic diagram of the optimal hTERT promoter-driven tyrosinase expression system.
